# Supplementary figures and images for: Contained Mycobacterium tuberculosis infection induces concomitant and heterologous protection
Source: PLoS Pathog. 2020 Jul 16;16(7):e1008655. doi: 10.1371/journal.ppat.1008655 (PMC7365393; doi:10.1371/journal.ppat.1008655)

S1 Fig

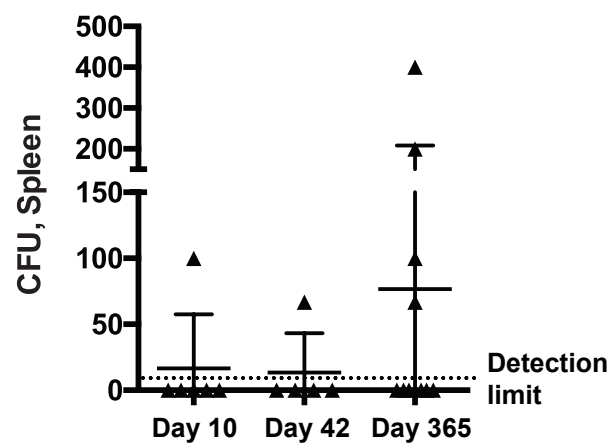

Supplement: S1 Fig — Mice were inoculated intradermally in the ear with 10,000 CFU of H37Rv and the bacterial burdens in the spleen and lung were measured at 10 days, 6 weeks, and 1 year by CFU assay (4–5 mice/timepoint). No bacteria were detected in the lung in any sample (detection limit 10 CFU / lung). (PDF) [file ppat.1008655.s001.pdf]

S2 Fig

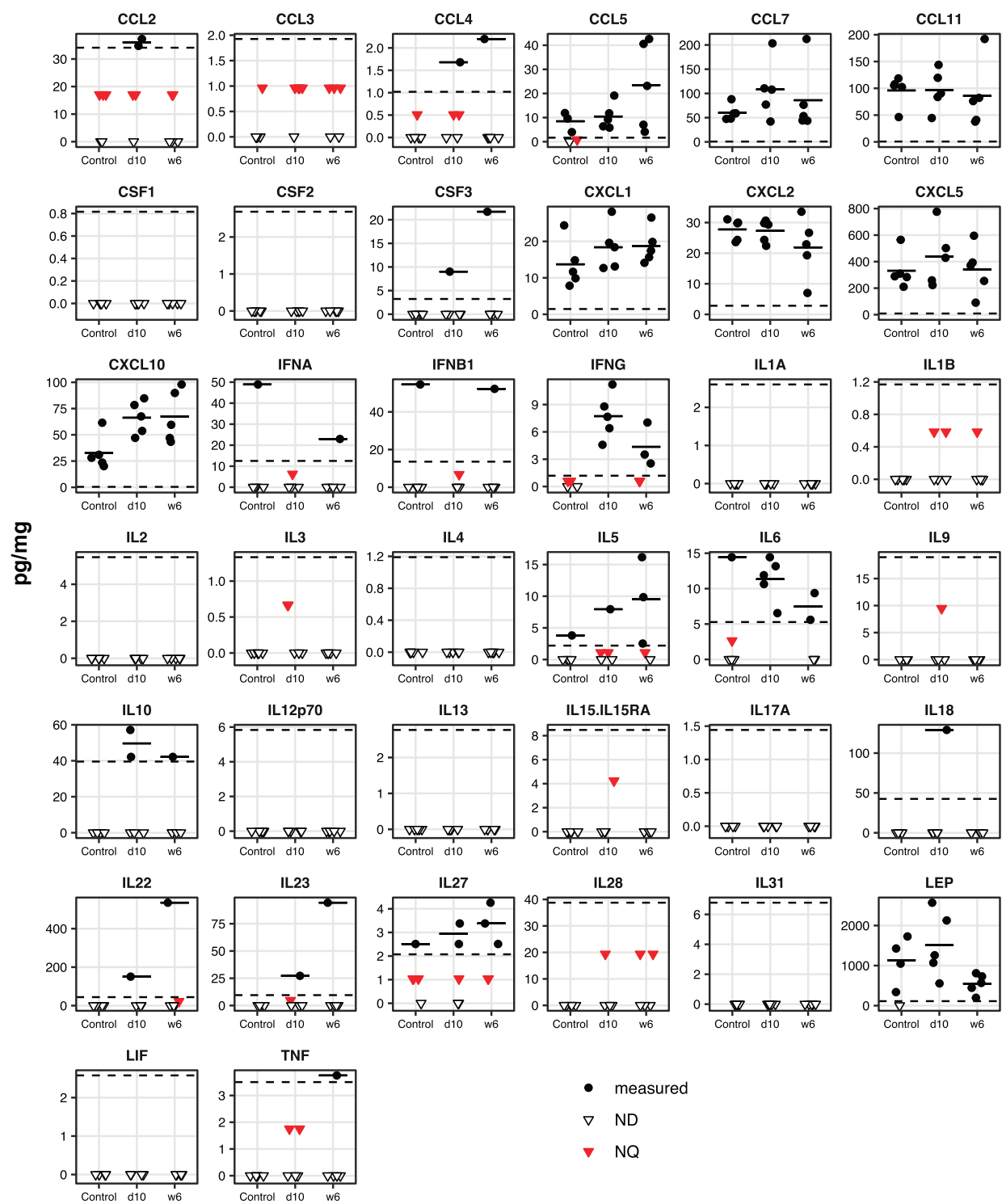

Supplement: S2 Fig — Blood was drawn from mice prior to or at 10 or 42 days following the establishment of CMTB and the serum concentrations of the indicated proteins measured by multiplexed immunoassay. Absolute levels of cytokines and chemokines normalized to the total protein in each sample are plotted. Horizontal lines indicate the mean values of measurements above the quantification limit. The quantification limit for each analyte is indicated with a dashed line. Measurements below the quantification limit are plotted with red markers at half of that value and measurements below the detection level are plotted with open markers. (n = 5 mice per condition) (PDF) [file ppat.1008655.s002.pdf]

S3 Fig

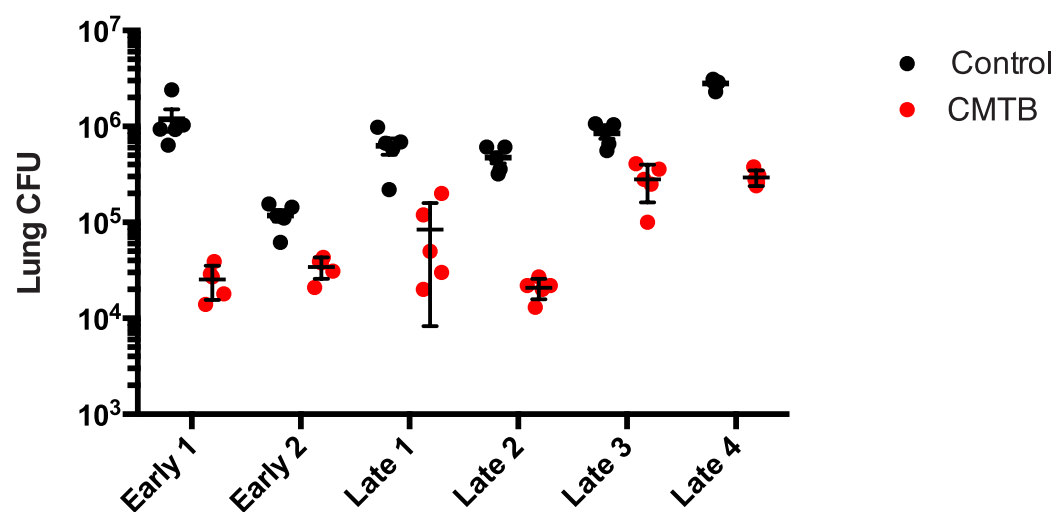

Supplement: S3 Fig — CMTB was established as described in the main text and mice challenged with 50–100 CFU of Mtb H37Rv via aerosol after 10–14 days (“Early”, 2 replicates) or after 8–10 weeks (“Late”, 4 replicates). Bacterial burden in the lung was measured by CFU assay. CMTB mice had on average 18.4-fold (CI: 10.6–26.3) fewer bacteria in the lung as compared to controls. In each individual experiment, the bacterial burden in CMTB mice was lower than that in control mice as determined by Student’s t-test (p < 0.05). Error bars depict mean and SEM. (n = 3–5 mice per group). (PDF) [file ppat.1008655.s003.pdf]

S4 Fig

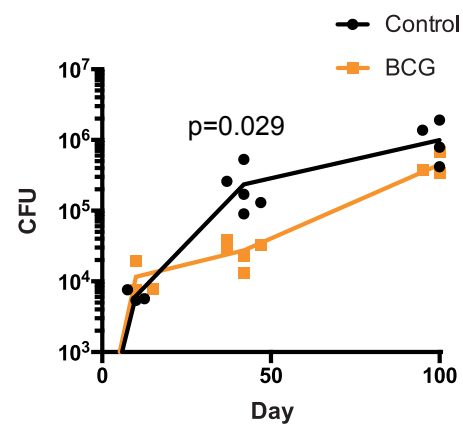

Supplement: S4 Fig — Mice were immunized sub-cutaneously with 1x10^6^ CFU BCG Pasteur and challenged with 100 CFU Mtb H37Rv via aerosol after 2 months. Bacterial burden in the lung was measured by CFU assay at days 10, 42, and 100 following aerosol challenge (n = 4–5 mice per group). Statistical significance was determined by Student’s t-test. (PDF) [file ppat.1008655.s004.pdf]

S5 Fig

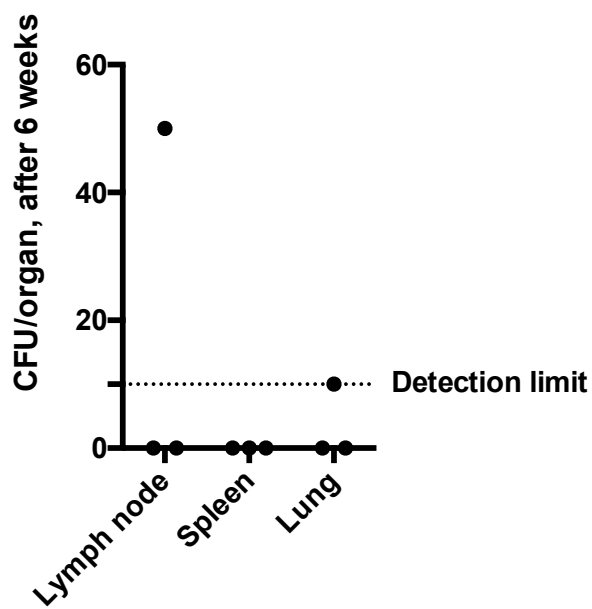

Supplement: S5 Fig — CMTB was established in mice and after 2 weeks mice were treated for 6 weeks with Isoniazid (0.1 μg/mL) and Rifampicin (0.1 μg/mL) in the drinking water. After treatment, mice were switched to untreated water for at least 1 week to allow complete clearance of the antibiotics from the mice. (The half-lives of Isoniazid and Rifampicin have been measured to be 4 hours and 2.5 hours). Three mice were sacrificed, and undiluted tissue homogenates of the cervical lymph nodes, lungs, and spleens were plated for CFU measurement. No CFUs were detected after the standard 3-week incubation period. The plot shows CFUs measured after 6 weeks of incubation. (PDF) [file ppat.1008655.s005.pdf]

S6 Fig

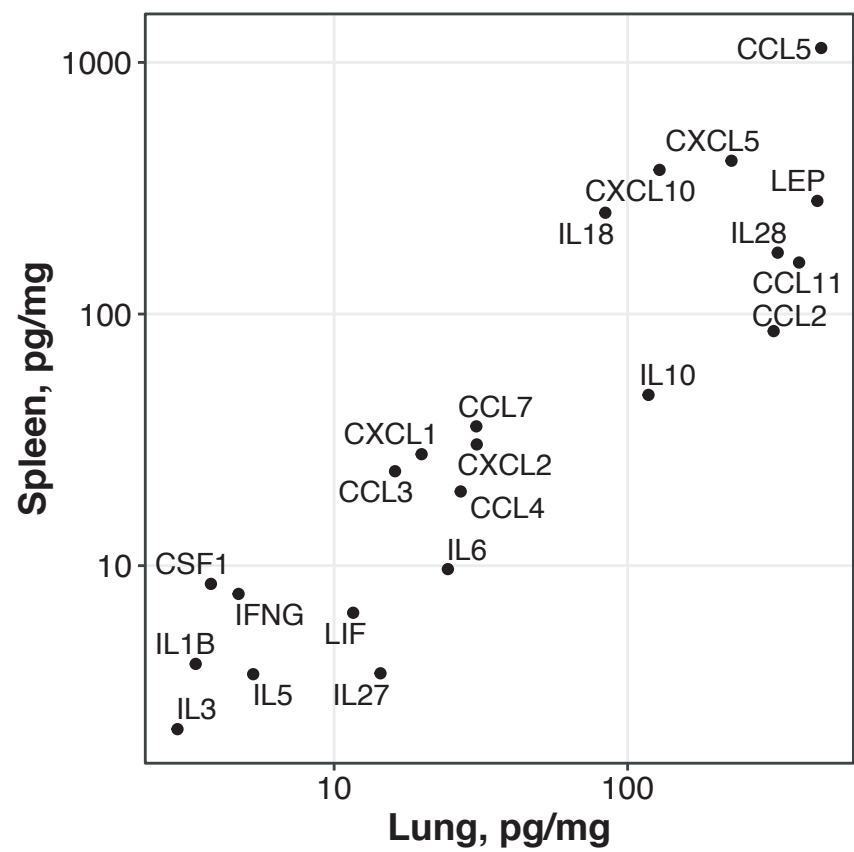

Supplement: S6 Fig — CMTB was established as described in the main text and the abundances of selected cytokines and chemokines in the lungs and spleen measured by multiplexed immunoassay at day 42 following inoculation. The plot depicts the levels of analytes that were detected in both tissues. Each point represents the average of 5 mice. (PDF) [file ppat.1008655.s006.pdf]

S7 Fig

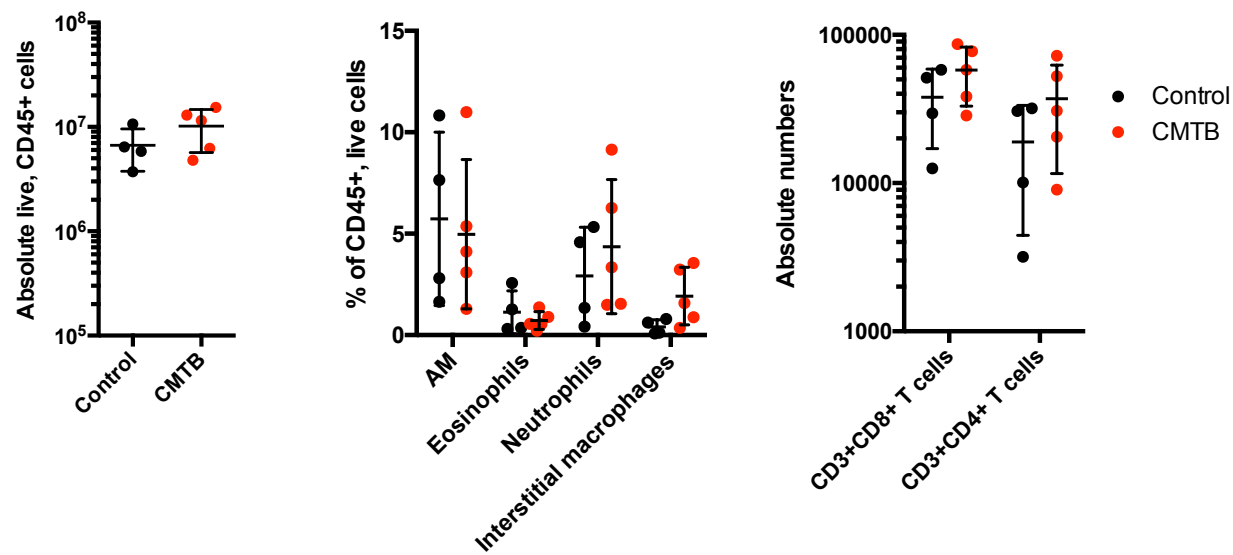

Supplement: S7 Fig — CMTB was established as described in the main text. At 8 weeks, Left panel: the absolute number of CD45+ cells in whole-lung homogenates was measured by flow cytometry using counting beads; Middle panel: The relative proportions of various immune cell populations were measured by flow cytometry. (See S17 Fig for gating.); Right panel: The absolute numbers of CD4+ and CD8+ T cells were measured by flow cytometry using counting beads. (See S14 Fig for gating.) Data are representative of two independent experiments with 4–5 mice per condition. (PDF) [file ppat.1008655.s007.pdf]

S8 Fig

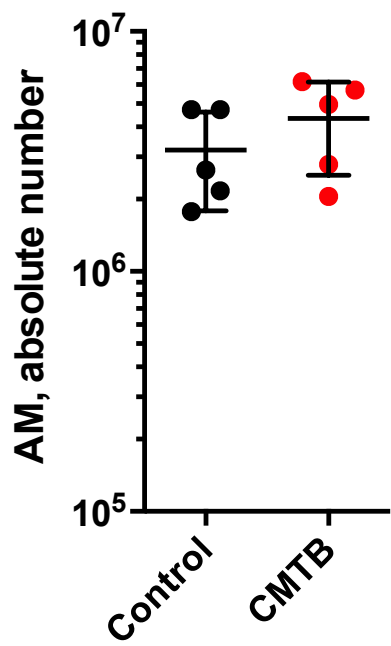

Supplement: S8 Fig — Absolute numbers of alveolar macrophages (CD11bintCD11c+CD64+Siglec-F+) in control and CMTB mice at 10 days following aerosol infection with 50–100 CFU of Mtb H37Rv. Error bars depict the mean and SD. Representative data from one of two independent experiments with 4–5 mice/group/timepoint. (See S17 Fig for gating strategy.) (PDF) [file ppat.1008655.s008.pdf]

S9 Fig

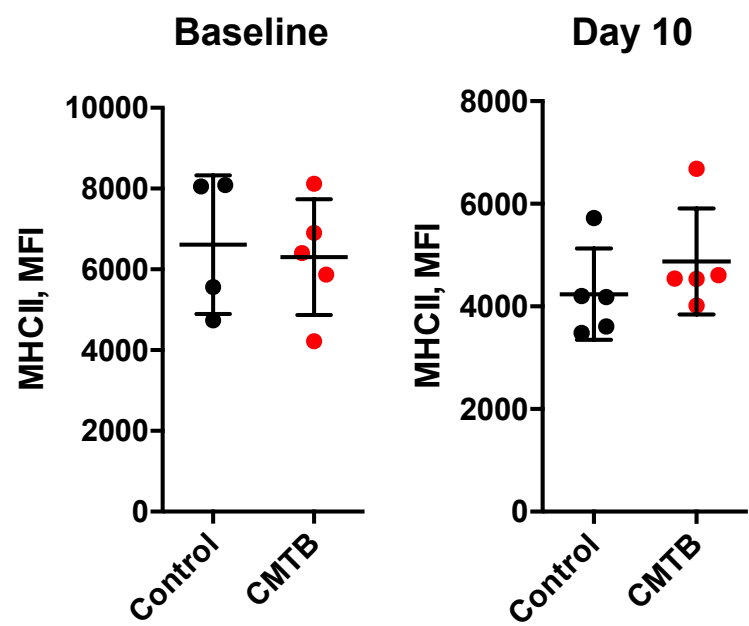

Supplement: S9 Fig — MFI of MHCII expression on CD11b+CD64+Siglec-F- monocytes extracted from control or CMTB mice at 10 days following aerosol infection with 50–100 CFU of Mtb H37Rv. Error bars depict the mean and SD. Representative data from one of two independent experiments with 4–5 mice/group/timepoint. (See S17 Fig for gating strategy.) (PDF) [file ppat.1008655.s009.pdf]

S10 Fig

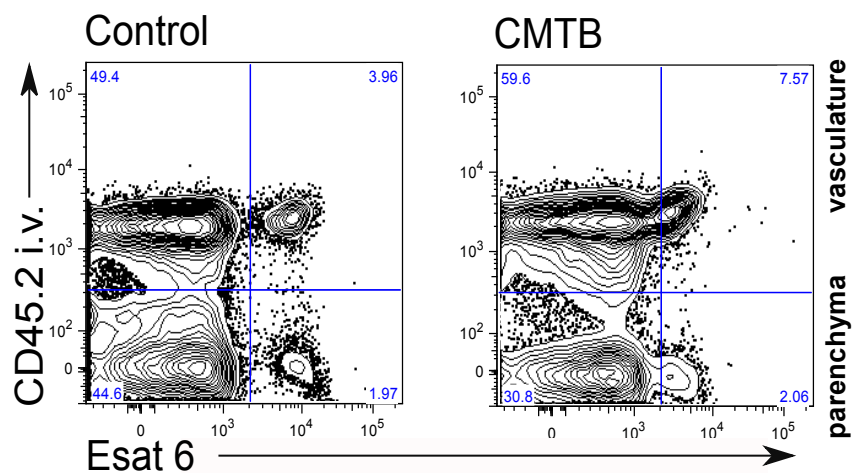

Day 10

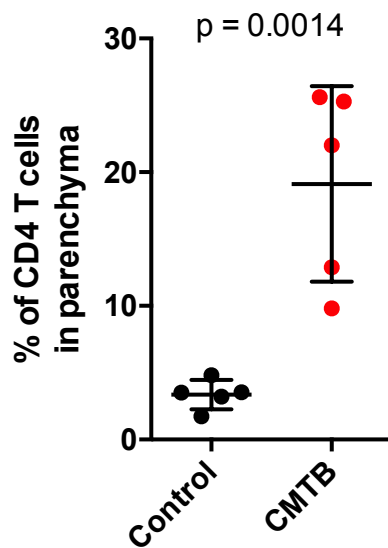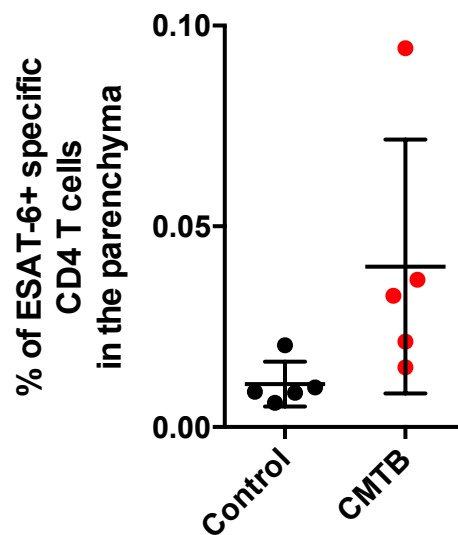

Day 42

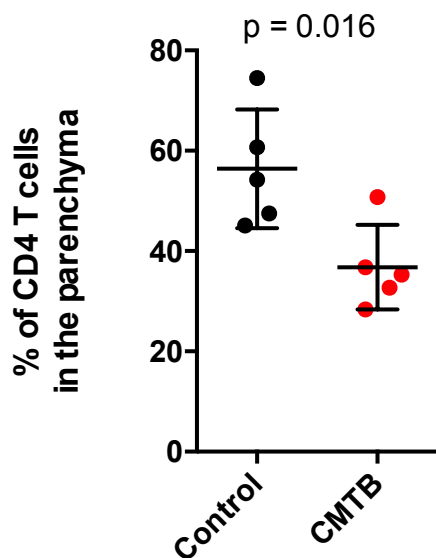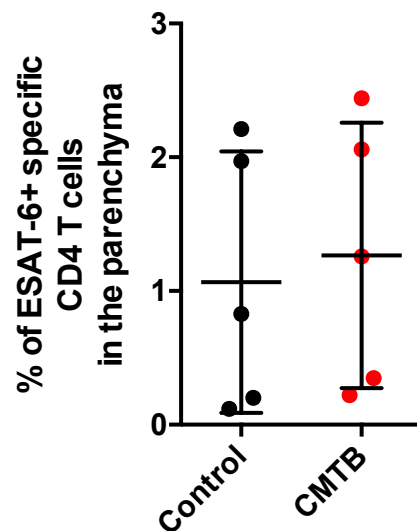

Supplement: S10 Fig — Quantification of total and Esat-6-specific CD3+CD4+ cells and in the lung parenchyma (CD45.2-PE-) in control and CMTB mice at 14- and 42-days following aerosol infection with 100 CFU of Mtb H37Rv. A representative cytometry plot of CD3+CD4+ cells is shown. Statistical significance was determined by Student’s t-Test. Error bars depict the mean and SD. Representative data from one of two independent experiments with 4–5 mice/group/timepoint. (See S14 Fig for gating strategy.) (PDF) [file ppat.1008655.s010.pdf]

S11 Fig

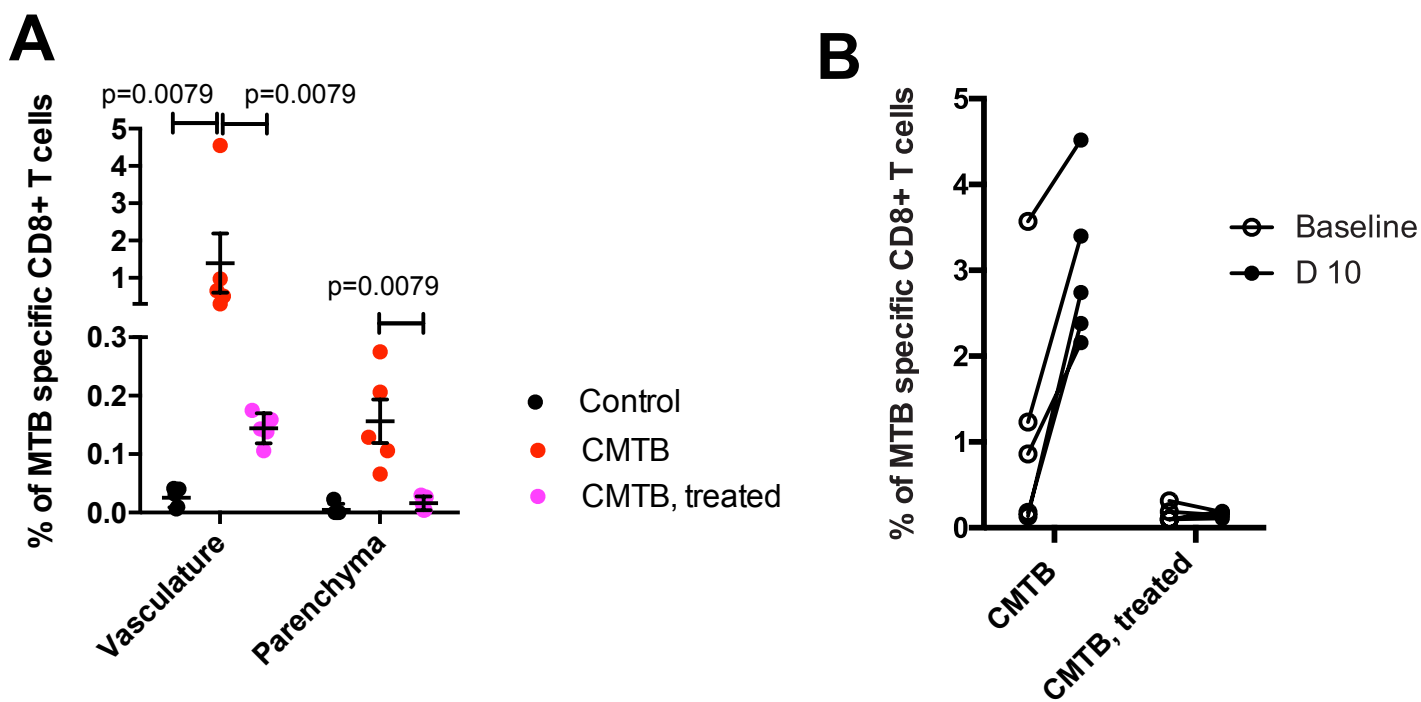

Supplement: S11 Fig — (A) Following antibiotic treatment to clear CMTB as described in the main text, the fraction of CD3+CD8+TB10.4+ T cells in the lung vasculature and parenchyma, as determined by i.v. labeling with anti-CD45-PE antibody was measured by flow cytometry 10 days following aerosol challenge with 50–100 CFU of Mtb H37Rv. Statistical significance was determined by Student’s t-Test. Error bars represent the mean and SEM (B) The fraction of CD3+CD8+TB10.4+ T cells in whole lung homogenates of CMTB and control mice prior to aerosol challenge with 50–100 CFU of Mtb H37Rv and at 10 days following challenge was determined by flow cytometry. Statistical significance was determined by paired t-test. Error bars depict the mean and SEM. (n = 3–5 mice per group). (See S14 Fig for gating strategy.) (PDF) [file ppat.1008655.s011.pdf]

S12 Fig

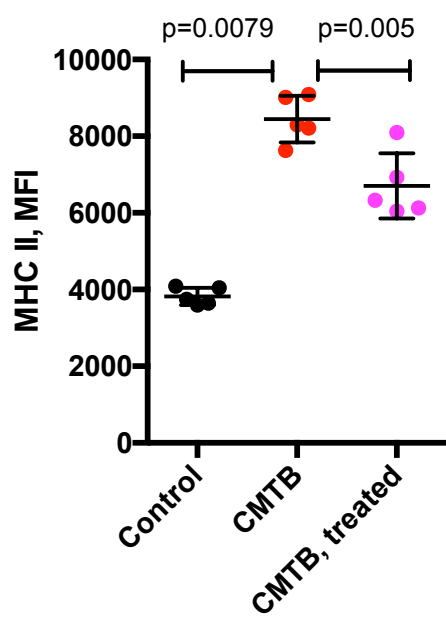

Supplement: S12 Fig — Flow cytometry analysis of alveolar macrophages from control, CMTB, and antibiotic-treated CMTB mice isolated 14 days following aerosol challenge with Mtb H37Rv. CD11bintCD11c+CD64+Siglec-F+ were gated on CD11b and Siglec-F to define AMs (see S17 Fig). MHC II expression was quantified by MFI. Statistical significance was determined by Student’s t-Test. Error bars represent the mean and SEM. (PDF) [file ppat.1008655.s012.pdf]

# S13 Fig

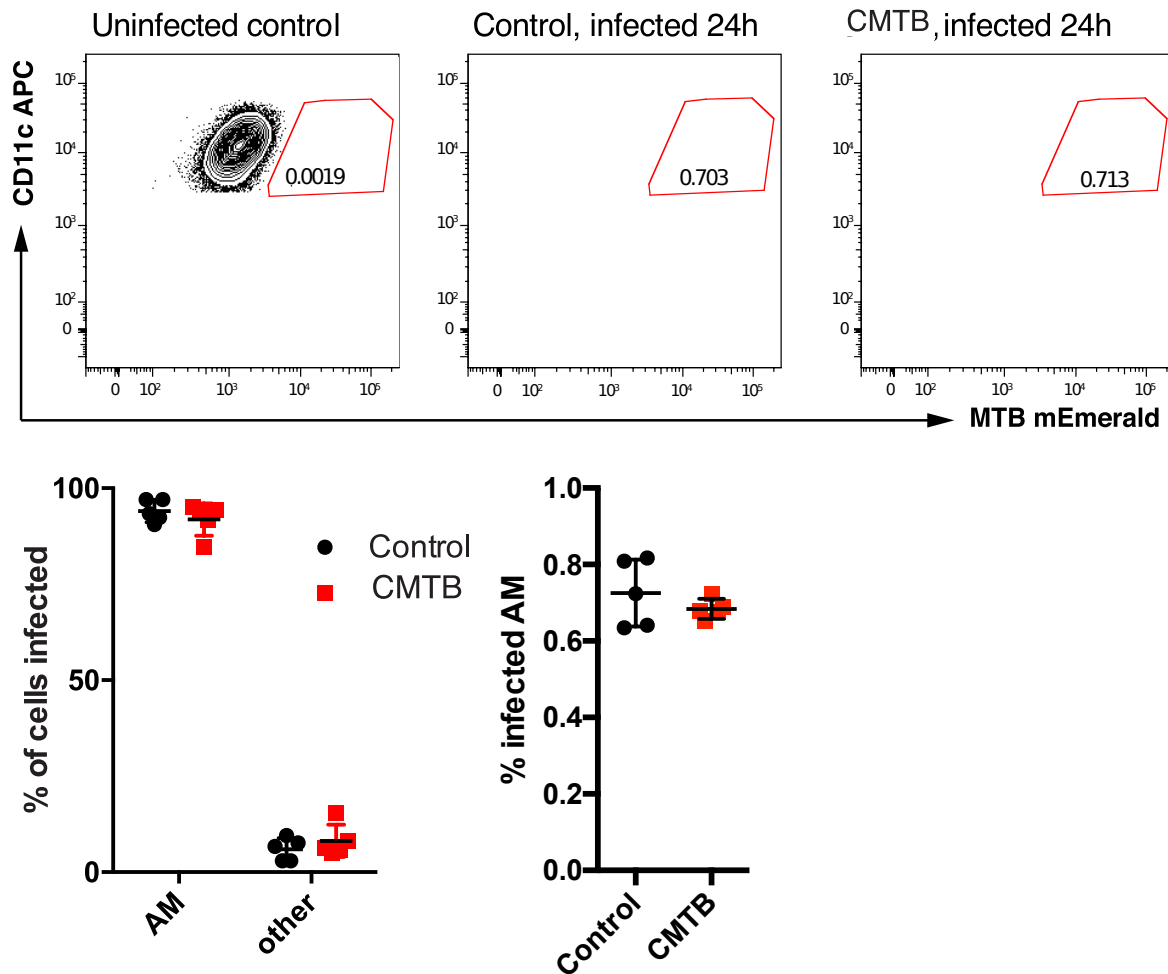

Supplement: S13 Fig — CMTB and control mice were infected with ~2000–4000 CFU of mEmerald-expressing Mtb via aerosol and the cellularity of bronchoalveolar lavage (BAL) fluid extracted 24 hours following infection was analyzed by flow cytometry. Top panels: Flow cytometry plots showing mEmerald+ Mtb-infected AMs gated on CD45+CD11bintCD11c+CD64+SiglecF+ cells (see S17 Fig for gating strategy). Bottom panels: Distribution of infected cell types (left) and fraction of AMs infected (right) in BAL fluid at 24 hours following high-dose infection (See Methods). Error bars depict the mean and SD. (PDF) [file ppat.1008655.s013.pdf]

S14 Fig

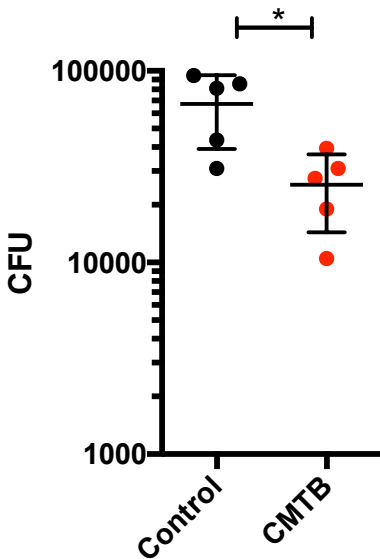

Supplement: S14 Fig — Control and CMTB mice were infected with ~2000–4000 CFU of Mtb and bacterial burden in the lung measured by CFU assay 10 days following challenge. Data are representative of 2 independent experiments with 4–5 mice per condition. Significance was assessed by Student’s t-test. Error bars represent the mean and SD. (PDF) [file ppat.1008655.s014.pdf]

S15 Fig

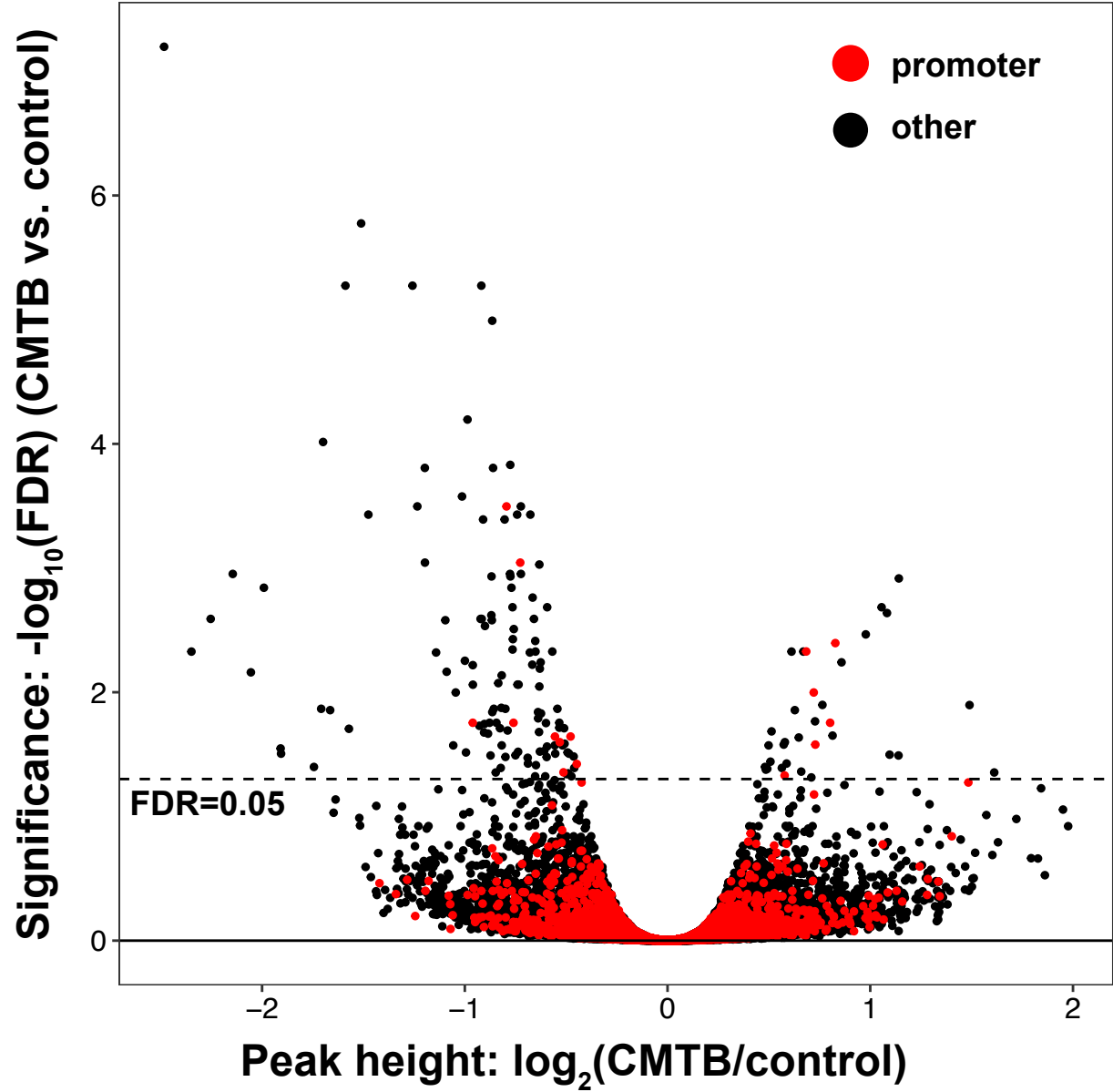

Supplement: S15 Fig — Alveolar macrophages from CMTB and control mice (n = 3/condition) were isolated from BAL fluid by FACS and ATAC-seq was performed following a published protocol [55]. Plot depicts FDR vs. difference in chromatin accessibility between control and CMTB for 45,458 genomic regions. Red dots indicate peaks within gene promotor regions. (See Methods). (PDF) [file ppat.1008655.s015.pdf]

# S16 Fig

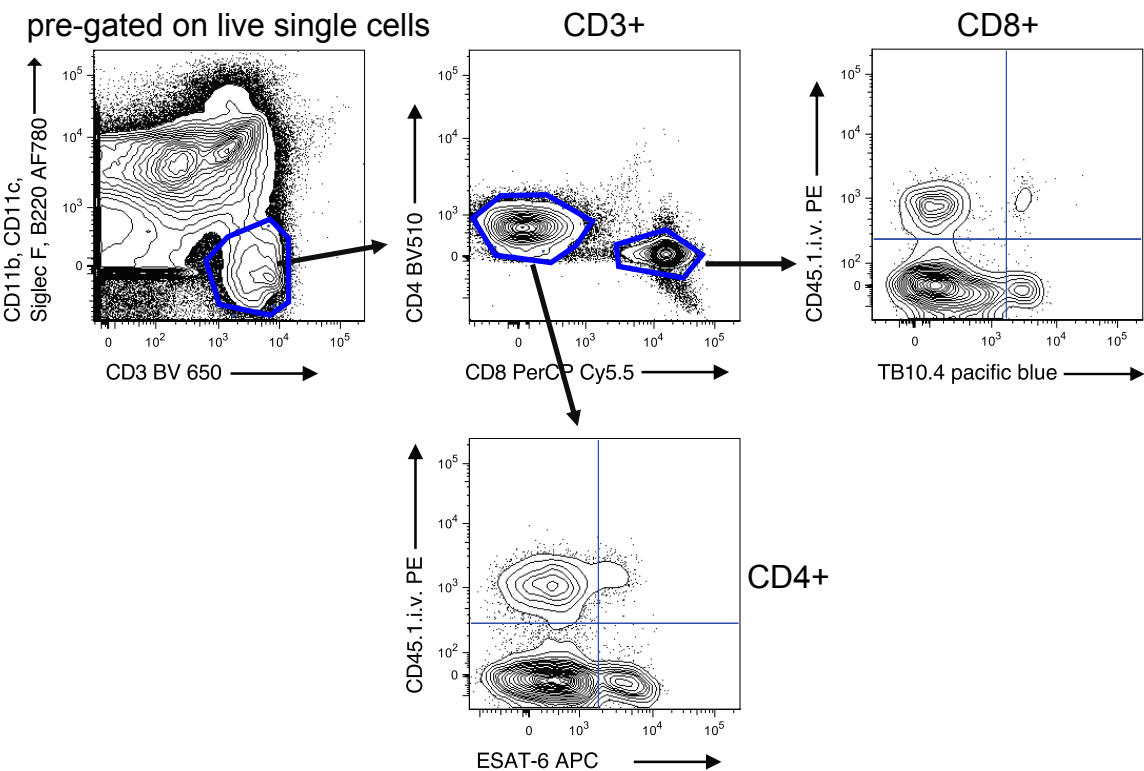

\*Siglec F was not used for peripheral blood stainings

Supplement: S16 Fig — Live (Zombie Violet-) single cells were gated on CD3, excluding CD11b+, CD11c+, Siglec-F+, and B220+ cells to define T cells and then on CD4 and CD8. For lung samples, localization of T cells to the lung parenchyma or vasculature was determined by i.v. labeling with and anti-CD45.1 antibody. (PDF) [file ppat.1008655.s016.pdf]

S17 Fig

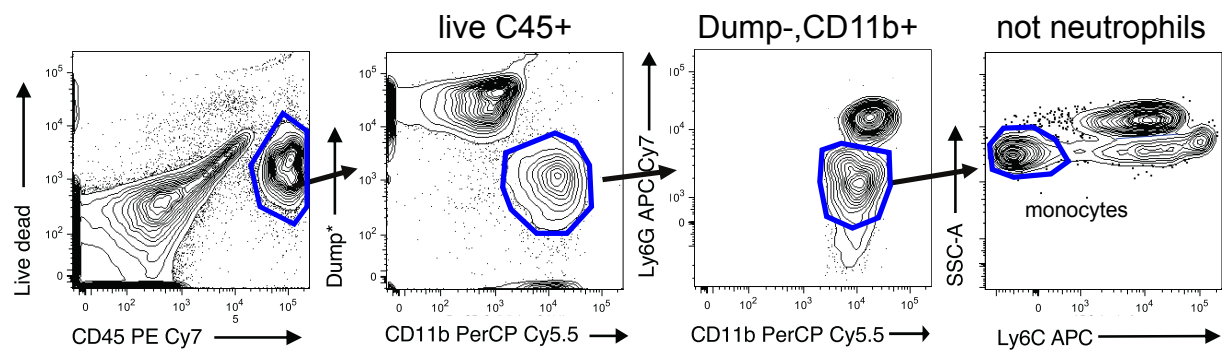

\*B220, CD3, NK1.1,CD11c - PE

Supplement: S17 Fig — Live (Zombie Violet-) single cells were gated on CD45, excluding B220+, CD3+, NK1.1+ and then on CD11b+, followed by Ly6G-, followed by Ly6C- to define monocytes. (PDF) [file ppat.1008655.s017.pdf]

# S18 Fig

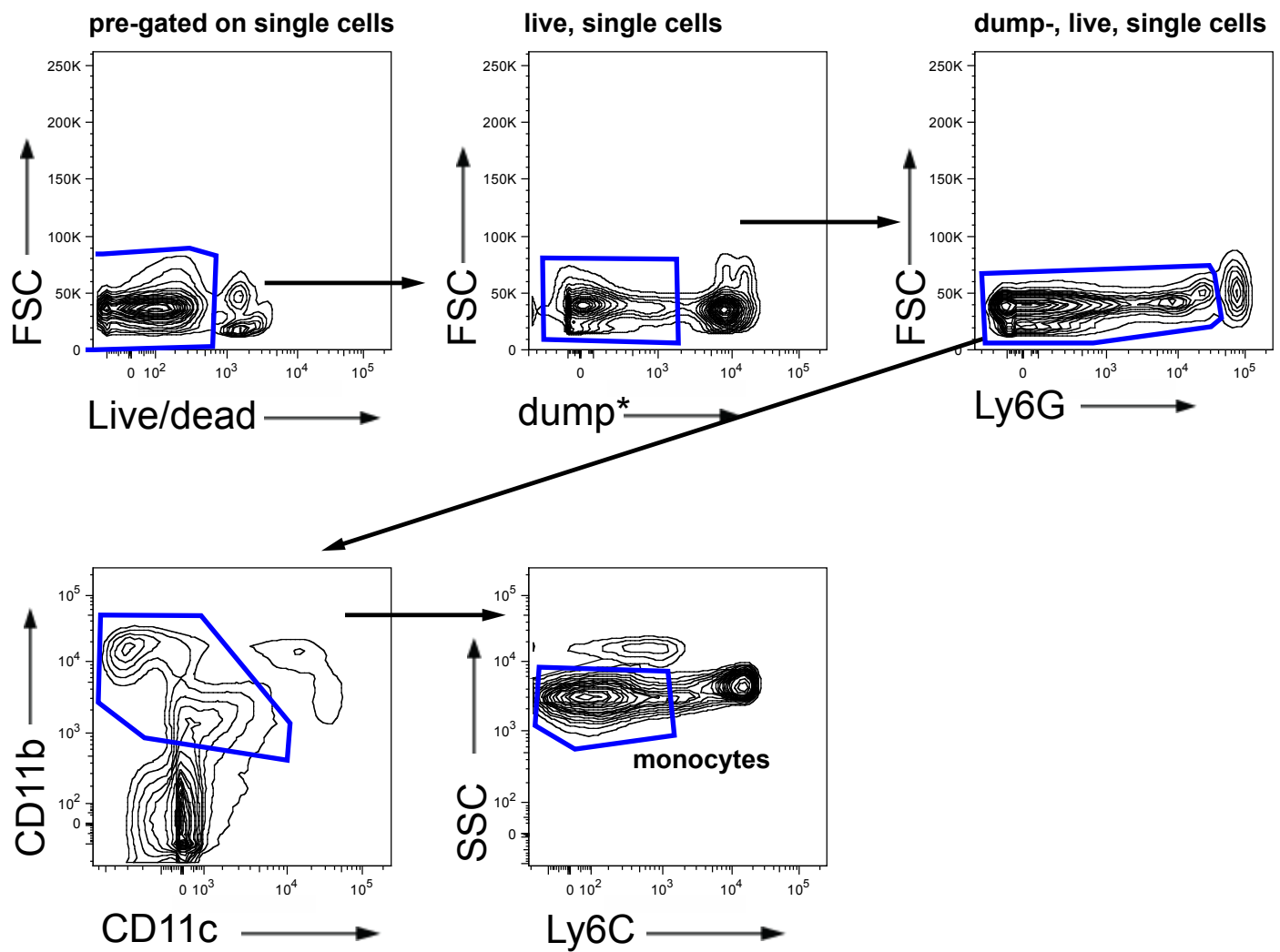

\*dump: CD3, B220, NK1.1

Supplement: S18 Fig — Live (Zombie Violet-) single cells were gated to exclude CD3+, B220, and NK1.1+. This population was then gated on Ly6Glow and then on CD11b+, CD11cint followed by Ly6c- to define monocytes. (PDF) [file ppat.1008655.s018.pdf]

# S19 Fig

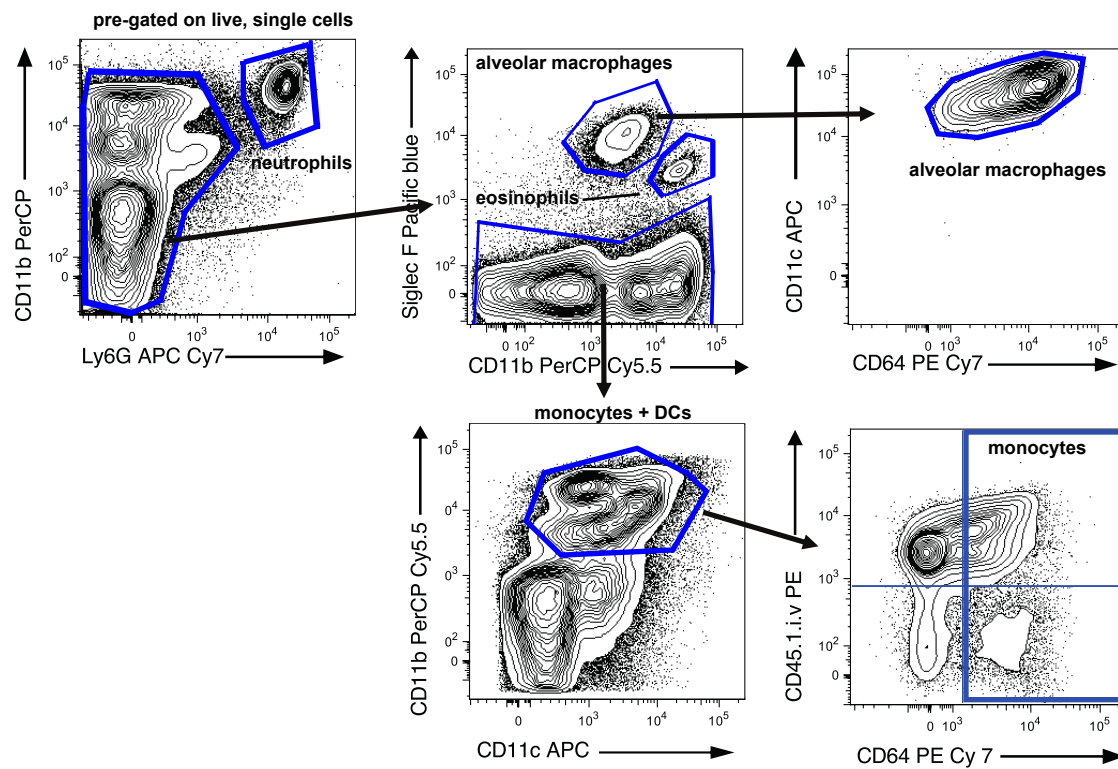

Supplement: S19 Fig — Live (Zombie Violet-) single cells were gated on CD11b and Ly6G to define neutrophils (CD11b+ Ly6G+). Ly6G- cells were further gated on Siglec-F and CD11b to isolate eosinophils (Siglec-F+ CD11bhigh), alveolar macrophages (Siglec-F+ CD11bmid), monocytes/interstitial maccrophages and dendritic cells (Siglec-F-). In order to robustly isolate alveolar macrophages in inflamed lungs, Siglec-F+ CD11bmid cells were further gated on CD11c and CD64. (PDF) [file ppat.1008655.s019.pdf]

# S20 Fig

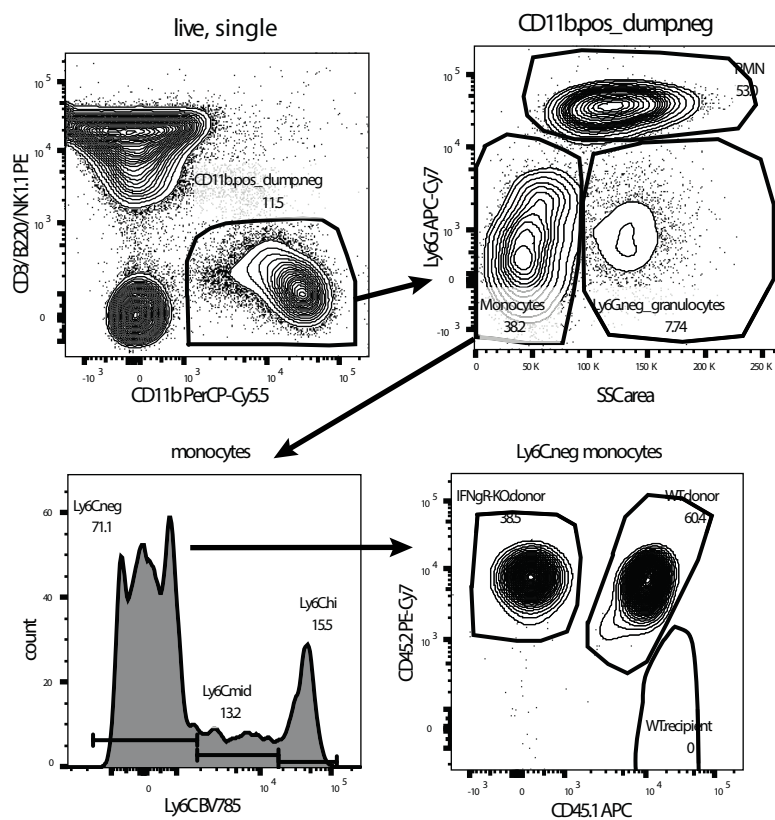

Supplement: S20 Fig — Live (Zombie Violet-) single cells were gated on CD11b+, excluding CD3, B220, and NK1.1. Monocytes were defined from this population as SSClow, Ly6G- cells and their genotypes assigned by CD45.1/2 labeling. (PDF) [file ppat.1008655.s020.pdf]

# S21 Fig

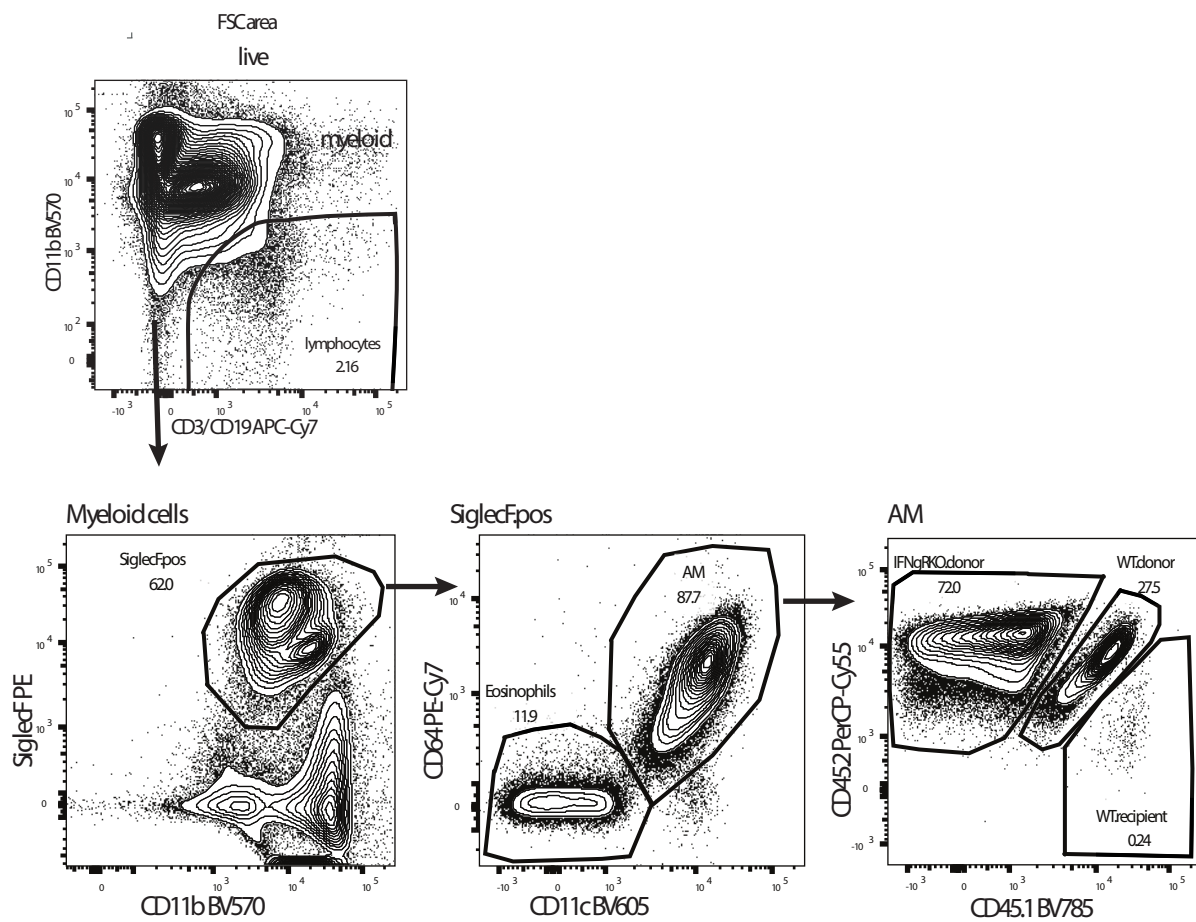

Supplement: S21 Fig — CD3+ and CD19+ cells were excluded from live, single cells to define the myeloid population. Alveolar macrophages were defined from this population as Siglec-F+, CD11c+, CD64+ cells and their genotypes assigned by CD45.1/2 labeling. (PDF) [file ppat.1008655.s021.pdf]
