# Supplementary material for: Contained Mycobacterium tuberculosis infection induces concomitant and heterologous protection
Source: PLoS Pathog. 2020 Jul 16;16(7):e1008655. doi: 10.1371/journal.ppat.1008655 (PMC7365393; doi:10.1371/journal.ppat.1008655)
Supplement: S1 Table — (PDF) [file ppat.1008655.s022.pdf]

	Extent 1	Extent 2	Mixed granulomas	Defined granulomas	PV LA*	PB LA**	Histiocytes	Foamy Macs	MNGC***	Neutrophils	Alveolar hyperplasia	Necrosis	Cholesterol clefts	Edema	AFB****	Total	comments
Control Day 14	1	1	0	0	0	0	0	0	0	0	0	0	0	0	1	**3**	multifocal minimal to mild accumulations of lymphocytes perivascular and within a vessel
Control Day 42	3	2	2	2	2	2	3	2	0	2	0	1	0	0	3	**24**	moderate acute multifocal hemorrhages
Control Day 100	3	3	3	2	2	3	3	3	0	2	0	1	1	1	3	**30**	moderate acute multifocal to coalescing hemorrhages
CMTB Day 14	3	2	2	2	2	2	2	1	0	1	0	0	0	0	1	**18**	
CMTB Day 42	2	2	1	1	2	1	2	1	0	0	0	0	0	0	2	**14**	
CMTB Day 100	2	2	1	3	3	1	3	3	0	0	0	0	0	0	1	**19**	

* PV LA: Perivascular lymphoid aggregates
** PB LA: Peribronchiolar lymphoid aggregates
*** MNGC (Multi-nucleated giant cells): clearly defined cell with > 3 nuclei
**** AFB (Acid-fast bacteria)

Table S1. Pathology analysis of CMTB mice
