# Supplementary material for: Contained Mycobacterium tuberculosis infection induces concomitant and heterologous protection
Source: PLoS Pathog. 2020 Jul 16;16(7):e1008655. doi: 10.1371/journal.ppat.1008655 (PMC7365393; doi:10.1371/journal.ppat.1008655)
Supplement: S2 Table — (PDF) [file ppat.1008655.s023.pdf]

Vendor	Product_Number	Description
BioLegend	103128	Alexa Fluor 700 anti-mouse CD45 antibody
BioLegend	100729	Alexa Fluor 700 CD8 antibody anti mouse
BioLegend	117310	APC anti-mouse CD11c Antibody
BioLegend	100516	APC anti-mouse CD4 Antibody
BioLegend	103112	APC anti-mouse CD45 Antibody
BioLegend	100712	APC anti-mouse CD8a Antibody
BioLegend	128015	APC anti-mouse Ly-6C Antibody
BioLegend	100221	APC/Cy7 anti-mouse CD3 Antibody
BioLegend	107627	APC/Cy7 anti-mouse I-A/I-E Antibody
BioLegend	128026	APC/Cy7 anti-mouse Ly-6C Antibody
BioLegend	115539	Brilliant Violet 605, anti-mouse CD19 Antibody
BioLegend	100237	Brilliant Violet 605, anti-mouse CD3 Antibody
BioLegend	100204	FITC anti-mouse CD3 Antibody
BioLegend	103108	FITC anti-mouse CD45 Antibody
BioLegend	137005	FITC anti-mouse CD68 Antibody
BioLegend	107605	FITC anti-mouse I-A/I-E Antibody
BioLegend	103006	FITC anti-mouse/human CD44 Antibody
BioLegend	108905	FITC CD49b NK cell anti mouse
BioLegend	100534	Pacific blue CD4 antibody anti mouse
BioLegend	103126	Pacific Blue, anti-mouse CD45 Antibody
BioLegend	104723	Pacific Blue, anti-mouse CD80 Antibody
BioLegend	103106	PE anti-mouse CD45 Antibody
BioLegend	103207	PE anti-mouse/human CD45R/B220 Antibody
BioLegend	115508	PE CD19 B cell anti mouse
BioLegend	117317	PE/Cy7 anti-mouse CD11c Antibody
BioLegend	109109	PE/Cy7 anti-mouse CD279 (PD-1) Antibody
BioLegend	100219	PE/Cy7 anti-mouse CD3 Antibody, 25ug
BioLegend	103114	PE/Cy7 anti-mouse CD45 Antibody
BioLegend	139313	PE/Cy7 anti-mouse CD64 (FcGRI) Antibody
BioLegend	123113	PE/Cy7 anti-mouse F4/80 Antibody
BioLegend	100538	PerCP anti-mouse CD4 Antibody
BioLegend	103130	PerCP anti-mouse CD45 Antibody
BioLegend	103132	PerCP/Cy5.5 anti-mouse CD45 Antibody
BioLegend	100733	PerCP/Cy5.5 anti-mouse CD8a Antibody
BioLegend	103031	PerCP/Cy5.5 anti-mouse/human CD44 Antibody
BioLegend	101320	TruStain (anti-mouse CD16/32) Antibody
BioLegend	423102	Zombie Aqua, Fixable Viability Kit
BioLegend	423114	Zombie Violet
Fisher Scientific L.L.C.	BDB557401	PE Hamster Anti-Mouse CD11c
Fisher Scientific L.L.C.	BDB552126	PE Rat Anti-Mouse Siglec-F
Fisher Scientific L.L.C.	BDB562419	PE-CF594 Rat Anti-Mouse CD127
Fisher Scientific L.L.C.	BDB550993	PerCP-Cy,5.5 Rat Anti-CD11b Clone M1/70 (RUO)
Thermo Fisher Scientific Life Technologies	47-0112-80	CD11b Monoclonal Antibody (M1/70), APC-eFluor 780, eBioscience
Thermo Fisher Scientific Life Technologies	17-0128-42	CD11c Monoclonal Antibody (BU15), APC
Thermo Fisher Scientific Life Technologies	63-0451-82	CD45 Monoclonal Antibody (30-F11), Super Bright 600
Thermo Fisher Scientific Life Technologies	MHCD4526	CD45 Monoclonal Antibody (HI30), Alexa Fluor 405
Thermo Fisher Scientific Life Technologies	MF48005	F4/80 Monoclonal Antibody (BM8), APC
Thermo Fisher Scientific Life Technologies	BDB562681	BV421 Rat Anti-Mouse Siglec-F Clone E50-2440 (RUO)
Thermo Fisher Scientific Life Technologies	L34957	LIVE/DEAD, Fixable Aqua Dead Cell Stain Kit
Thermo Fisher Scientific Life Technologies	56-5321-80	MHC Class II (I-A/I-E) Monoclonal Antibody (M5/114.15.2), Alexa Fluor 700, 25ug

Table S2. Antibodies used
