# Supplementary material for: Contained Mycobacterium tuberculosis infection induces concomitant and heterologous protection
Source: PLoS Pathog. 2020 Jul 16;16(7):e1008655. doi: 10.1371/journal.ppat.1008655 (PMC7365393; doi:10.1371/journal.ppat.1008655)
Supplement: Attachment — Submitted filename: Nemeth_etal_CMTB_Response_to_Reviews_final.docx [file ppat.1008655.s024.docx]

**Response to peer reviews from eLife. Reviewer comments are in blue and our responses are in black.**

Reviewer #1 (Dr. Joel Ernst, MD, Chief, Division of Experimental Medicine, UCSF) (General assessment and major comments (Required)): 

This manuscript reports results of extensive studies that reveal that a chronic, stable M. tuberculosis infection results after subcutaneous injection of bacteria in an ear, with bacteria residing in the local draining lymph node for at least one year. The studies further reveal that this chronic stable infection provides (modest) protection against an aerosol challenge of M. tuberculosis. The protection observed extends to systemic Listeria and to melanoma cells and is associated with a heightened state of activation of pulmonary alveolar macrophages; this heightened state depends on IFNgamma responsiveness. 

Overall, this is a valuable contribution, and is sure to prompt additional studies to extend the findings and determine their generalizability. If any criticism were to be raised, it would be that most of the results are descriptive, and the mechanistic insights are modest and limited to the findings that the effect of CMTB depends on IFNgamma responsiveness. In this reviewer's opinion, this is not a major limitation, since mechanistic studies need to be based on observational studies, and the quality and impact of those in this case is very high. 

Minor comments: 

Abstract: The comment, "An efficacious vaccine against adult tuberculosis remains elusive" should be updated to acknowledge results of the M72/ASO1E trial.

We have edited the abstract to acknowledge the results of the M72/ASO1E trial: “Progress in tuberculosis vaccine development is hampered by an incomplete understanding of the immune mechanisms that protect against infection with *Mycobacterium tuberculosis* (Mtb), the causative agent of tuberculosis. Although the M72/ASOE1 trial yielded encouraging results (54% efficacy in subjects with prior exposure to Mtb), a highly effective vaccine against adult tuberculosis remains elusive. We show that…”

p. 5, line 13; and Discussion, line 1: "....the mouse model..." should be restated to acknowledge that there is no single mouse model of TB. 

We have edited the two sections to read:

“Although antigen-specific T cell responses likely contribute to the protection afforded by CMTB against aerosol challenge in mice …”

and

“The mouse continues to play an essential role as a model organism for the development of TB vaccine strategies due to the extensive availability of molecular reagents, a large body of historical data, and relatively low cost [33]. Much of our current understanding of TB immunology had its origins in the mouse before subsequent validation in humans, for example the critical for CD4 T cells[34][1], IFNγ[35,36], IL-12[37], and TNF [38].”

The following comments relate to the Discussion: 

Cytokine levels in tissues: Page 11 lines 16 - 20; Could the authors give suggestions on how CMTB leads to cytokine elevation in other tissues (particularly lung) when the bacteria couldn't be detectable in this tissue after CMTB establishment? Are there plausible reasons to think that cells acquire signals in the cervical lymph node and then traffic to other tissues, or are the effects due to systemic effects of low concentrations of cytokines?

We added the following paragraph to the Discussion:

“It is experimentally challenging to define with certainty the mechanisms by which CMTB leads to elevated cytokine levels in tissues without detectable bacteria, such as the lung. Given that the total number of CD45^+^ cells in the lung does not change, that we measure elevated levels of inflammatory mediators such as IFNG, IL6, and CXCL10 in the blood, and that we observe increased activation of circulating innate immune cells, we believe it is likely that the increase in tissue cytokine levels arises from low grade cytokinaemia. However, we cannot exclude the possibility of a contribution from immune cells that were activated in the cervical lymph node and subsequently migrated to the lung.”

Difference between CD8 and CD4 antigen specific responses: Page 6 line 22 - 23; the authors could not detect ESAT-6 specific CD4 T cells in blood, lung and spleen in CMTB mice but CD8 antigen specific T cells were detected. Since other literature indicates that CD8 T cell responses are lower in magnitude and generally correspond to high bacterial populations, this is a surprising result. More discussion of this point would be valuable. 

In other TB studies using C57BL/6 mice, we have detected much greater numbers of TB10.4-specific CD8 T cells than ESAT-6-specific CD4 T cells in both the lung and draining lymph nodes during the first month following infection (For example, see Figure 7 in Shafiani et al., *Exp Med* (2010) 207 (7): 1409–1420.) As measured by total cell numbers, the ESAT-6-specific response begins to approach that of the TB10.4-response only by day 30 and only in the lung. Given the low-level of infection in the CMTB model, we were therefore not surprised that we were unable to detect ESAT-6 specific CD4 T cells prior to aerosol challenge. It is possible that they could be detected by tetramer enrichment and we intend to pursue this in future studies that will characterize the quality of the adaptive response to CMTB in greater detail. The relative magnitudes of the CD4 and CD8 responses are also mouse strain dependent. C57BL/6 mice do not mount a TB10.4-specifc CD4 response while those responses can be quite strong in BALB/c mice.

We added the following point to the discussion:

Although we easily detect circulating TB10.4-specific CD8+ T cells in CMTB mice prior to aerosol challenge we were unable to detect ESAT-6 specific CD4+T cells in any compartment. In other studies using C57BL/6 mice, we detected much greater numbers of TB10.4-specific CD8+ T cells than ESAT-6-specific CD4+ T cells in both the lung and draining lymph nodes during the first month following aerosol infection (Shafiani et al., *Exp Med* (2010) 207 (7): 1409–1420.). Given that the bacterial burden in lymph node in the CMTB model is much lower than that in the lung at 30 days following aerosol challenge (10^4^ CFU vs. 10^5^-10^6^ CFU) our results are consistent with previous measurements of the relative abundances of these T cell subsets induced by Mtb-infection.

On page 43 line 14 and supplemental figure 8, the total CD4 T cells in the parenchyma is reversed in magnitude in the lung parenchyma between Controls and CMTB mice at days 14 and 42, could this elevated total CD4 T cells in the lung parenchyma of control contributing to the pathology? Any explanation for this difference? 

We interpret these data as indicating that the early influx of CD4 T cells in CMTB mice helps to control early bacterial growth whereas the larger numbers of CD4 T cells in control mice at day 42 reflects the much higher bacterial burden at this time point and the longer time required to generate significant CD4 T cell responses in mice that have not previously been infected with TB.

Reviewer #1 (Minor Comments): 

Other comments 
Reference 1 is a 2016 report, a more recent report (and the corresponding numbers) should be cited. 

We have updated this reference and numbers to cite the WHO Global Tuberculosis Report from 2019.

Can any information be gotten from existing flow cytometry files regarding the activation status of innate lymphoid cells in the lungs of CMTB vs control mice?

Unfortunately, we didn’t include markers for innate lymphoid cells in our flow-cytometry panel.

Page 11 line 22; not all cytokines were low in the tissue as they report in the preceding paragraph (unless the authors refer to the blood). Suggestion is to rewrite the last part of line 22 to capture the results already presented.

We have re-written this sentence to read: “Although the composition of the myeloid cell populations in the lungs of CMTB and control mice were not significantly different (Figure S7), we hypothesized that their function might be affected by elevated cytokine levels in CMTB mice.”

Page 12 third last line, change CD11bintCD11c+CD64+Siglec-F+ to CD11bintCD11c+CD64+Siglec-F+ 

We have corrected a couple of typos in the figure caption.

Page 16 the Venn diagrams (in page 15) legends need labels too for ease of reference

We have added labels to the Venn diagrams in this figure.

Page 16 figure E (what of the white bars?) 

We have edited the caption to note that the white bars label pathways there were not enriched with (FDR > 0.05) in the indicated condition.

Page 16 figure F add a scale for the heatmap 

We have added a scale to the heatmap.

Page 16 figure G comment on the PMNs that have more bacteria in CMTB than the control mice. 

We have added the following sentence to the text: “We also measured a corresponding increase in the numbers of infected polymorphonuclear leukocytes (PMNs) in CMTB mice, suggesting an accelerated cellular response compared to controls.”

Page 17 line 13; the x-axis (panel 2) has COTB instead of CMTB 
Fixed.

Page 18 line 4; larger faction, change to larger fraction 
Fixed.

Page 42 line 19; the authors report no CFUs yet there is a data point at around 50-mark for Lymph node on the graph (page 49), could the authors clarify this.

As stated in the caption, we detected no CFUs on plates incubated for **3 weeks** which is the standard incubation period for determining the presence of viable Mtb. In order to stringently test for the presence of bacteria that may attenuated by antibiotic treatment, we continued to incubate the plates for an **additional 3 weeks**. At this time point, which as stated in the caption is plotted, we measured 50 CFUs in one lymph node and 10 CFUs in one lung (the detection limit). We interpret these results as indicating that at most, only very small numbers of highly attenuated bacteria were present following Isoniazid/Rifampicin treatment.

Page 43 line 21 and supplemental figure 9; label the figures as A and B to match the legend description and for the second panel, change COTB to CMTB.

Fixed.

Page 51 Day 42 figure (left panel); change CMTB symbols to squares from circles to match the rest of the symbols for this group

Fixed.

Supplemental Figure 15: why are Ly6Chi monocytes excluded?

For these measurements, which are presented in Fig. 5A (with the gating strategy described in Fig. S15), we wished to examine the “tissue resident macrophages” in peripheral blood and not macrophages recently recruited from the bone marrow. Because circulating Ly6C low monocytes are generally accepted to be long-lived and sensitive to blood-cytokine levels, we consider them to be a good comparison to Alveolar Macrophages from the lung.

Reviewer #1 (Additional data files and statistical comments): 

The data files provided are sufficient. 


Reviewer #2 (General assessment and major comments (Required)): 

This manuscript describes an interesting, if non-physiological, infection model for Mycobacterium tuberculosis (Mtb) called the "Contained Mtb" or CMTB infection model. In this model, mice are infected intradermally (in the ear) with virulent Mtb. This results in long-lasting infection of cervical lymph nodes and modestly elevated systemic inflammation but does not result in disease, as the infection remains mostly contained. Aerosol lung challenge of the mice with virulent Mtb results in about 10-25-fold lower bacterial burdens in the lungs as compared to naive mice. Protection is also seen against heterologous challenges, such as iv Listeria or melanoma, suggesting that innate immunity provides an important component of protection. Ongoing infection seems important to provide protection as antibiotic treatment of mice reverses the protective effects of CMTB. The authors propose that the systemic inflammation, particularly the production of systemic IFN gamma, that arises from CMTB acclerates early innate and adaptive immune responses in the lung 

Overall the manuscript is well-written and the data are clearly presented and described. The phenotype of CMTB mice is characterized thoroughly, using cutting-edge experimental approaches. However, I am not convinced that the impact of these findings is sufficient for eLife. The degree of protection seen in this model is better than conventional BCG, but is still relatively modest. Correlates of protection are identified, but everything is just a correlation, and despite being a mouse model, few causal relationships are established. So while the authors claim that their mouse model affords the opportunity to do mechanistic immunology that is impossible in other systems, in the end, it is not entirely clear why CMTB provides protection (what changes seen in these mice actually provide protection versus which changes are just correlating with protection). The changes that are seen are what would be expected. My feeling is that is little here that is conceptually new that would significantly move the needle for the field. 

Reviewer 2 feels that the impact of this study is likely to be less than Dr. Ernst suggests based on three arguments: 1) the mouse model of Mtb-infection is “non-physiological”, 2) the degree of protection induced by CMTB is “modest”, and 3) the present study does not provide sufficient insight into the protective mechanisms.

By definition, any animal model is “non-physiological” compared to humans. Nevertheless, countless fundamental insights into the immunology and pathology of TB disease have arisen from studies in animal models including non-human primates, rodents, and even fish. Much of our current understanding of TB immunology had its origins in the mouse before subsequent validation in humans. For example, the critical roles for CD4 T cells, IFNγ, IL-12, and TNF. The mouse continues to be an essential model organism for the development of TB vaccine strategies due to the extensive availability of molecular reagents, a large body of historical data, and relatively low cost.

It is widely believed that asymptomatic TB encompasses a variety of disease states, often referred to as the “spectrum of TB disease”, including bacterial clearance, persistence of live bacteria contained by the immune system, and sub-clinical disease. In both humans and non-human primates, the lymphatic system plays key roles in the spectrum of disease by providing a reservoir for latent Mtb and coordinating subsequent immune responses.

Following acute TB disease in humans, the bacterial burden often dramatically contracts and is maintained at a very low level either in pulmonary granulomas or in the draining lymph nodes and the patient is asymptomatic. In contrast, in mice the bacterial burden in the lung following the standard 50-100 CFU aerosol challenge rapidly increases to a high level and is relatively constant until death. Therefore, in the aerosol challenge model, it is impossible to study the role of Mtb contained in the lymphatic system during an asymptomatic phase of infection. By delivering Mtb directly to the cervical lymph node, the CMTB model circumvents this limitation and enables studying in mice an important portion of the disease spectrum in humans. In fact, the model was originally developed by Dr. Stefan Kaufmann to study emergence from latency arising from immunocompromise as is seen in humans infected with HIV or on immunosuppressive therapies.

Reviewer 2 argues that the protection afforded by CMTB against aerosol challenge is “modest”. However, as described in the paper and in our point-by-point comments, CMTB gives protection that is 1) at least as effective as sub-cutaneous BCG at day 42, the time-point at which this “gold-standard” in mice shows maximum protection, 2) effective in reducing bacterial growth 10-fold as early as 1o days following infection (a time-point at which BCG is ineffective), and 3) more durable than BCG (10-fold protection at day 100, a time-point at which BCG is no longer effective). To our knowledge, the protective effect of CMTB is as strong and durable as that of any vaccination strategy which has been demonstrated in mice. Given the importance of the mouse in the study of TB immunology, we argue that a protection modality this powerful is likely to provide significant insights.

Finally, Reviewer 2 notes the absence of exhaustive studies to fully disentangle the mechanisms underlying the protection arising from CMTB. This is the first paper to describe the protective effect of CMTB in mice. As Dr. Ernst points out, “… mechanistic studies need to be based on observational studies, and the quality and impact of those in this case is very high” and that this work, “… is sure to prompt additional studies…”. In fact, under the IMPAc-TB program ($30 million in first-year funding), the NIH recently funded three large research contracts that take a cross-species approach to defining the correlates of protection against TB disease. All of these projects rely heavily on the mouse and one will mechanistically dissect the CMTB model.

The contemporary and historical evidence suggests that this work will have significant impact and we strongly disagree with Reviewer 2’s “feeling”.

Reviewer #2 (Minor Comments): 

Other minor comments 
- is it unexpected that there is no induction of a detectable CD4 T cell response in CMTB mice? is this a sensitivity problem that might be solved with tetramer enrichment?

Please see response above to a similar question from Reviewer 1.

- recent work has suggested that the development of resident memory T cells in lung is important to mediate protection against Mtb. In this system, is the modest degree of protection due to the fact that T cells are in the vasculature and not in the parenchyma prior to challenge? Could protection be enhanced by a strategy to draw the induced T cells into the correct tissue?

While it is certainly possible that stronger protection could be achieved by a vaccination approach that was able to draw greater numbers of Mtb-specific T cells into the lung prior to aerosol challenge, developing, testing, and speculating about such a strategy is beyond the scope of this paper.

Although the level of protection afforded by CMTB could be considered “modest” relative to the goal of a vaccine potent enough to achieve bacterial clearance in mice following a 50-100 CFU aerosol infection, no vaccine strategy has been demonstrated that comes within several logs of this goal. To our knowledge, the 10-20-fold protection afforded by CMTB is as strong, rapidly acting, and durable as any that has been demonstrated in the mouse (See reference 32 in the main text.).

- it is difficult to understand why there is no transcriptional difference in AMs from control vs CMTB mice (prior to infection), given that there are apparently changes at the level of cell surface proteins (e.g., MHC class II). The data in Fig 5C strongly suggests these cells are responding to systemic IFNg, so why aren't transcriptional changes seen? Is there a sensitivity issue? 

Reviewer 2’s statement that “there is no transcriptional difference” is incorrect. If we apply stringent criteria for differential expression (|log_2_(fold-change)| > 1 and FDR < 0.05, corresponding to a 2-fold difference and a 1 in 20 chance of a false positive, as stated in the caption) only a small number of genes meet these criteria. Therefore, based on these parameters, which are typical when examining genome-wide mRNA express, we stated that the transcriptomes “did not differ substantially” which accurately characterizes the subtle effect of CMTB on AMs prior to infection. If those criteria are relaxed, a greater number of genes would be called as “differentially expressed” but the biological difference between the two samples is obviously unchanged.

Note that even if protein synthesis directly tracked mRNA expression, the 60% average increase in MHCII expression on AMs would not have been called as “significant” under the criteria defined above, despite being easily measured by flow cytometry. Therefore, the transcriptomic and flow cytometry measurements are consistent and indicate a subtle but detectable effect of CMTB on AMs prior to aerosol challenge.
