# Supplementary material for: Contained Mycobacterium tuberculosis infection induces concomitant and heterologous protection
Source: PLoS Pathog. 2020 Jul 16;16(7):e1008655. doi: 10.1371/journal.ppat.1008655 (PMC7365393; doi:10.1371/journal.ppat.1008655)
Supplement: Attachment — Submitted filename: Nemeth_et_al_CMTB_ResponseLetter.docx [file ppat.1008655.s025.docx]

May 22, 2020

Dr. JoAnne Flynn, Section Editor,

Dr. Christopher Sassetti, Associate Editor

Dr. Kasturi Haldar, Editor-in-Chief

Dr. Michael Malim, Editor-in-Chief

Dear Drs. Flynn, Sassetti, Haldar, and Malim,

We thank the reviewers for their careful reading of the manuscript and suggestions for improvement and clarification. We have included each point from the reviews below in blue and inserted our responses in black. Modifications to the text are referred to by line numbers in the revised manuscript.

**Part I - Summary**
Please use this section to discuss strengths/weaknesses of study, novelty/significance, general execution and scholarship.

Reviewer #1: In this manuscript entitled ‘Contained Mycobacterium tuberculosis infection induces concomitant and heterologous protection’, Nemeth J et al have demonstrated that after subcutaneous (Ear) Mtb infection, the bacteria persisted in the draining lymph node for one year and generated protection in mice re-exposed to Mtb via aerosol route. Interestingly, Mtb infection in the ear also provided heterologous protection against systemic Listeria infection as well as melanoma. The protection in CMTB mice was linked to the low level of circulating IFN-γ, activated alveolar macrophages (higher expression of MHCII), and the recruitment of Mtb-specific T cells to the lung parenchyma. The reviewer agrees with the initial assessment of Dr. Ernst that this is an important study and provides more insights to natural immunity against TB. However, the conclusion of the current study needs to be solidified by additional experiments.

Reviewer #2: In this manuscript, Nemeth et al., describe a new model for chronic, low-grade Mtb infection in mice that is established after injection of Mtb bacilli into the ear and is maintained for more than a year. This chronic, low-grade infection induces low-level systemic inflammation that appears to activate alveolar macrophages and that can protect against reinfection with Mtb by aerosol challenge. This effect, which was abrogated in the absence of IFNgR, demonstrating that it is dependent on IFNg signaling, extended beyond mycobacteria and also protected against Listeria and melanoma cells.
The paper reports a novel and interesting model and the first insights into the possible mechanistic underpinnings of concomitant immunity in Mtb, which may be analogous to that observed in humans with controlled, quiescent Mtb infection. The manuscript is well written and the findings are illustrated clearly and are easy to follow. I do not have any major concerns beyond those raised by the previous round of reviews for eLife, which I think were addressed well. The paper may be improved by addressing some minor points.

Reviewer #3: In this manuscript, Nehmes and colleagues describe a chronic ear infection model of Mtb that allows the authors to assess the impact of chronic contained infection on subsequent pulmonary exposure. While not perfectly similar to human “latent” infection, this represents a creative attempt to expand the suite of animal models of tuberculosis.

This manuscript was submitted with reviews from ELife where it seems to have been rejected on the basis of Reviewer 2’s critique that the work was not sufficiently novel or mechanistic. I strongly disagree with this assessment. There is no equivalent small animal model. Moreover, as Dr. Ernst says as Reviewer 1, novel observations from well executed studies are a critical foundation for subsequent mechanistic work –and probably on average hold up better over time.

I do have series of more granular concerns of which the final one is the most serious:

-Low dose challenge model: I have concerns about the analysis. Protection is manifested as reduction in “take” –the number of bacteria that infect where the answer can be 0,1,2 or 3 and subsequent growth/control of those bacteria, which is exponential-ish. First, the methods say that the authors excluded counts from mice that had no CFU. I am not sure that it is legal to exclude the zeros and it obscures biology where showing in up in 1 vs 2 lobes might be an effect on take or spread.

We have re-written and clarified the text describing the ULD experiments in the Results (page 7), Methods (page 18-19)), and the caption to Figure 2 (page 33). As described in the modified text, under the assumption that there is no clearance of bacteria below the detection limit, the effect of CMTB on susceptibility to infection (the “take” in the reviewer’s term) is separable from the effect on control of bacterial replication. The fact that the fraction of infected mice is very similar between days 42 and 102 in both control and CMTB mice supports the assumption of negligible bacterial clearance. We find that CMTB does not affect susceptibility to infection but strongly reduces bacterial burden. We have also clarified the analysis and interpretation of the measurements of bacterial dissemination to the opposite lung following ULD challenge.

-Heterologous immunity: I disagree with the interpretation of the Leishmania and melanoma studies. I think that these studies indicate that CMTB provides heterologous and perhaps innate protection against these two challenges but it does not necessarily follow that the protection against Mtb is through the same mechanism and likely to be innate.

We assume that the reviewer is referring to the *Listeria* experiments; we did not perform any studies with Leishmania for this paper.

While we agree that the demonstration of heterologous protection against *Listeria* and melanoma challenges does not prove a role for innate immunity in CMTB-mediated protection against Mtb, it does indicate that CMTB significantly enhances innate responses. The experiments showing elevated transcriptional and functional responses in AMs are strongly suggestive of an innate immune contribution. These cells are the first to encounter Mtb in the lung and are the critical first step for instructing adaptive responses, whether naïve or primed.

It is unlikely that CMTB, or any immune-modulating intervention, confers a single protective mechanism against Mtb-infection. Because T cells are required to maintain CMTB, cleanly separating the protective roles of innate and adaptive cells with a single experimental strategy is impossible. In fact, the IFNg which drives elevated activation of AMs in CMTB mice likely arises from Mtb-specific T cells and therefore the distinction between “innate” and “adaptive” mechanisms in this system is somewhat artificial.

We have edited the text (pages 8, and 15) to emphasize that CMTB likely protects against Mtb-infection through multiple mechanisms.

-Unappreciated T cell responses: The authors seem to favor innate mechanisms of control but their data strongly suggest the contribution of a primed T cell response and less convincing makes a case for myeloid memory. Indeed, I am not sure why the authors do not think the differences that they see in AM transcriptional responses post challenge (the IFN signature) do not reflect the consequences of a primed T cell response. Similarly, I am not sure how pure the sorted AM populations were but a little T cell contamination with some associated Ifng production could explain the apparent differences in AM ability to restrict Mtb (4H)

Nowhere in the manuscript do we use the term “myeloid memory”, which generally refers to changes in innate cells that persist after an infection has been eliminated and that modify responses to subsequent challenges. Our data demonstrate that the protective effect of CMTB depends on the presence of live, contained Mtb (pages 6-7). In addition, because we do not observe substantial epigenetic changes in AMs, our data suggest that CMTB does not induce “trained immunity” in these cells.

We measure amplified AM responses to Mtb at 24 hours following infection. Given the negligible numbers of Mtb-specific T cells in the lung parenchyma in CMTB mice (Fig. 1C), we believe it is unlikely that direct T cell – AM interactions can explain the elevated AM responses measured *in vivo*.

Given that elevated activation of AMs depends on IFNg, and that IFNg is detectable in the circulation of CMTB mice and plausibly arises from T cells in the cervical lymph node, one could say that amplified AM responses to Mtb formally arise from primed T cell responses. However, we believe that our data do not support a significant role for direct T cell-AM interactions at 24 hours following infection.

The *ex vivo* stimulation experiments (Fig. 4H) were performed with AMs isolated by brochoalveolar lavage. Lymphocytes account for less than 1% of live, CD45^+^ cells in these samples prior to further purification by washing of non-adherent cells. Cellularity measurements of whole lung samples from CMTB mice (Fig. 1C) indicate that only a very small percentage of T cells susceptible to extraction by BAL (those not in the lung vasculature) are Mtb-specific and therefore potentially producing IFNg. Based on these considerations, we consider it extremely unlikely that these experiments are confounded by IFNg produced *ex vivo*.

**Part II – Major Issues: Key Experiments Required for Acceptance**
Please use this section to detail the key new experiments or modifications of existing experiments that should be absolutely required to validate study conclusions.

Generally, there should be no more than 3 such required experiments or major modifications for a "Major Revision" recommendation. If more than 3 experiments are necessary to validate the study conclusions, then you are encouraged to recommend "Reject".

Reviewer #1: 1) The authors have provided tantalizing data of how innate immunity in particular AM play an important role in protection against pulmonary Mtb in CMTB mice. However, the cellular mechanisms of cross-protection of CMTB in listeria or melanoma models have not been addressed. If the authors assume that potentially the bone marrow derived monocytes/macrophages in CMTB were providing better protection in these model systems, can they simply assess the inflammatory capacity of BMDM (CMTB vs Control) in ex-vivo model of PAMPs stimulation (Similar to Figure 3B). Moreover, the authors can also treat these BMDM in vitro with a low dose of IFN-g to recapitulate the potential impact of low circulating IFN-g on these cells and their protection capacity against subsequent Mtb or listeria infection (in vitro CFU).

2) While the investigators suggest that IFN-g signaling was required for the activation marker (MHC-II) of monocytes and AM in mixed BM chimeras, the impact of this reduction on protection against Mtb was not demonstrated. What is the level of CFU in the lungs/spleens of these chimeric mice after Mtb infection? It is also intriguing that the pathway analysis reveals type I IFN (IFN-a) was highly induced in CMTB (Figure 4E). Can the authors perform the same mixed BM chimeras using IFNAR KO mice? The comparison of type I vs type II would be of interest in this model system. Hence several studies suggest that type I IFN is detrimental in immunity to TB, while it has been shown that IFN-a play a key role in regulating macrophages-mediating immunopathology in influenza viral infection (PMID:31110361).

3) As all the human BCG vaccination is intradermally, the study will benefit from comparison of CMTB to intradermal (Ear) BCG vaccination (CBCG!) to see how potentially a virulent strain vs an avirulent strain may affect trained immunity against subsequent pulmonary Mtb infection.

Reviewer #2: (No Response)

Reviewer #3: above

We appreciate the reviewer’s thoughtful input on further experiments that would mechanistically dissect the protective mechanisms conferred by CMTB in greater detail. In fact, we are taking many of these approaches in our future studies. Based on the editor’s comment that, “Some new experiments were suggested to further define mechanism. However, upon balancing the reviews, the editors believe that all reviewer critiques can be addressed with revisions to text, analyses and conclusions.”, we have not responded here to the specific suggestions from Reviewer 1.

**Part III – Minor Issues: Editorial and Data Presentation Modifications**
Please use this section for editorial suggestions as well as relatively minor modifications of existing data that would enhance clarity.

Reviewer #1: 1) The vast majority of LTBI have been initially infected with Mtb via pulmonary route and thus their AM are not “naïve” and have been exposed to Mtb. In fact, this comparison is significantly weakened their central hypothesis as the authors indicated that the presence of Mtb in the skin draining lymph nodes and not lung initiates a strong immunity to pulmonary Mtb infection. Thus, the reviewer highly encourages the authors of not comparing this model system (CMTB) to latent TB infection in humans.

We have edited the Discussion (page 17) to emphasize this important difference between latent TB in humans and the CMTB model.

2) Can the authors provide some information of inflammatory responses at the initial site of infection (ear)?

We have not observed any obvious inflammatory response in the dermis of the ear following inoculation. The bacteria are rapidly trafficked to the cervical lymph node and generally undetectable in the ear tissue after 5 days.

3) Alveolar macrophages from CMTB mice showed higher expression of MHC II. Did the author study the expression of MHCII on other CD11b+ cells in CMTB mice both pre and post aerosol Mtb infection? It’s interesting to assess the expression of MHCII or other activation marker on CD11b+ cells.

We found that expression of MHCII on CD11b^+^CD64^+^Siglec-F^-^ pulmonary monocytes was equivalent in control and CMTB mice both prior to and following aerosol challenge with Mtb. We have edited the text to include this information (page 9) and included the data as Supplemental Figure 9.

4) CMTB mice showed more recruitment of monocytes derived macrophages in the lungs 14 days post Mtb aerosol infection. Is there any change in the frequency or total AM in the lungs pre and post aerosol Mtb infection?

We did not measure any differences between control and CMTB mice in terms of the absolute numbers or percentages of AMs in the lungs prior to aerosol challenge (Fig. S7). Likewise, the absolute number of AMs was the same for both control and CMTB mice at 10 days following aerosol infection. We have added this information to the text (page 9) and included it as Supplemental Figure 8.

5) What is the source of IFN-γ in the lung of CMTB mice?

Determining the source of the circulating and pulmonary IFNg in CMTB mice is one of the focuses of our future experiments. Our current hypothesis is that most IFNg arises from Mtb-specific T cells in the cervical lymph node that are containing the infection. We have added this point to the Discussion (page 15).

Reviewer #2: 1. The results of statistical analyses are reported as asterisks that represent categories of p-values. It would be more appropriate if the reader can interpret these results based on the actual p-value. As such, wherever possible the authors should show actual p-values (even when these are above 0.05, or whatever threshold was used) instead of asterisks.

We have modified all of the figures and supplementary figures to show the actual p-values rather than asterisks.

2. The authors claim that their results provide insights into new vaccine development approaches, but do not discuss how their results can be used to advance this field. The authors should augment their discussion by adding some text about how the findings can be used to improve vaccination approaches.

We have expanded the Discussion (pages 13-14) to emphasize specific aspects of the CMTB model that are most relevant to vaccine development, such as protection that, in contrast to BCG, is both durable and affects bacterial burden early after infection.

Reviewer #3: above

Sincerely,

Alan Diercks, Ph.D.	Alan Aderem, Ph.D.

Sr. Scientist, SCRI	Professor, SCRI
